# Automated fluorescence lifetime imaging plate reader and its application to Förster resonant energy transfer readout of Gag protein aggregation

**DOI:** 10.1002/jbio.201200185

**Published:** 2012-11-27

**Authors:** Dominic Alibhai, Douglas J Kelly, Sean Warren, Sunil Kumar, Anca Margineau, Remigiusz A Serwa, Emmanuelle Thinon, Yuriy Alexandrov, Edward J Murray, Frank Stuhmeier, Edward W Tate, Mark A A Neil, Chris Dunsby, Paul M W French

**Affiliations:** 1 Institute of Chemical Biology, Department of Chemistry, Imperial College LondonSouth Kensington Campus, London, SW7 2A, UK; 2 Photonics Group, Department of Physics, Imperial College LondonSouth Kensington Campus, London, SW7 2AZ, UK; 3 Department of Chemistry, Imperial College LondonSouth Kensington Campus, London, SW7 2AZ, UK; 4 Retroscreen Virology LtdLondon, EC1 2AX, UK; 5 Pfizer Worldwide Research and DevelopmentPfizer Limited, Sandwich, Kent, CT13 9NJ, UK; 6 Centre for Histopathology, Imperial College LondonDu Cane Rd, London, UK

**Keywords:** fluorescence lifetime imaging microscopy (FLIM), FRET, HIV-1 Gag, HCA, protein-protein interactions

## Abstract

Fluorescence lifetime measurements can provide quantitative readouts of local fluorophore environment and can be applied to biomolecular interactions via Förster resonant energy transfer (FRET). Fluorescence lifetime imaging (FLIM) can therefore provide a high content analysis (HCA) modality to map protein-protein interactions (PPIs) with applications in drug discovery, systems biology and basic research. We present here an automated multiwell plate reader able to perform rapid unsupervised optically sectioned FLIM of fixed and live biological samples and illustrate its potential to assay PPIs through application to Gag protein aggregation during the HIV life cycle. We demonstrate both hetero-FRET and homo-FRET readouts of protein aggregation and report the first quantitative evaluation of a FLIM HCA assay by generating dose response curves through addition of an inhibitor of Gag myristoylation. *Z* ′ factors exceeding 0.6 are realised for this FLIM FRET assay.

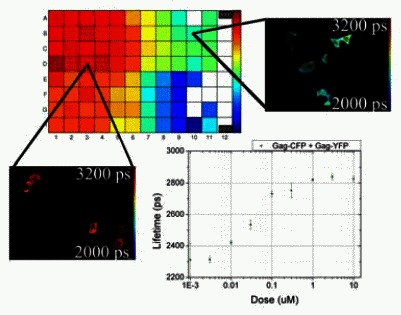

Fluorescence lifetime plate map with representative images of high and low FRET cells and corresponding dose response plot.

## 1. Introduction

The trend towards automated high content assays, particularly for systems biology and drug discovery, has stimulated the development of automated fluorescence microscopy systems to image sample arrays, e.g. in microtitre plates [1, 2]. Today's commercially available HCA instruments predominantly employ fluorescence intensity imaging in one or more spectral channels to map the relative distribution and co-localization of labelled proteins with subcellular resolution. There is increasing interest in harnessing fluorescence-based readouts for protein-protein interactions (PPIs) [3] with FRET measurements now commonplace in biology laboratories. However, to date, this has not been widely taken up for drug discovery. FRET entails exciting a “donor” fluorophore and observing the consequences of a transfer of the excitation energy to an appropriate “acceptor” fluorophore. This energy transfer is mediated by a direct dipole–dipole interaction and only occurs between fluorophores separated by less than ∼10 nm [4], enabling the detection and observation of binding of appropriately labelled proteins, either as end points in fixed cells or as dynamic events in live cells. FRET can also be used to read out conformational changes of a single molecule labeled with both donor and acceptor fluorophores and this is the basis of a range of genetically expressed intracellular biosensors that enable *in vivo* readouts of analytes such as calcium [5], potassium [6] and chloride [7] ions and signalling molecules such as IP_3_ [8], PIP_2_ [9] and calpain [10], among others [11]. Imaging techniques that can map molecular interactions by reading out FRET are therefore desirable to reveal the spatio-temporal organization of biomolecular interactions within cells, for example during cell signalling. Unfortunately conventional (manual) FRET microscopy is too time-consuming and labour-intensive for high throughput applications, e.g. screening libraries of reagents or siRNA gene knockdowns, and so the development of practical HCA FRET instrumentation is desirable for drug discovery and basic research.

While there are many approaches to map FRET [12], the most widely used are spectral ratiometric imaging and FLIM. The former can be easier to implement on existing instrumentation, requiring fewer detected photons and providing faster imaging than FLIM, but the latter can provide more quantitative readouts and requires less in the way of control/calibration samples. Spectral ratiometric FRET suffers from uncertainties associated with relative donor/acceptor concentrations, the spectral response – including the instrument and the sample itself (inner filter effect) – and cross-talk (e.g. direct excitation of the acceptor and spectral bleed-through in the detection channels). These issues are mitigated when using single molecule FRET biosensors (with known stoichiometry) to follow dynamics (i.e. relative changes) but quantitative FRET measurements require additional control samples, labelled with donor only and acceptor only, to calibrate the system. Spectral ratiometric FRET measurements can then yield the relative proportion of donor and acceptor molecules and the effective FRET efficiency (i.e. the product of the actual FRET efficiency and the fraction of FRETing donor/acceptor molecules) [13]. To obtain the fractions of the FRETing donor and acceptor populations, it is necessary to independently determine the actual FRET efficiency – either by measuring further (positive control) samples or by using other methods, such as acceptor photobleaching or FLIM.

Polarisation resolved anisotropy imaging is an alternative ratiometric technique to map FRET that exploits the decrease in fluorescence anisotropy of the acceptor in the presence of FRET [14]. This technique can reach similar imaging speeds to spectral ratiometric imaging and has recently been implemented in an automated multiwell plate reader [15]. While it provides high contrast for detecting FRET, it is also sensitive to spectral cross-talk (e.g. direct excitation of the acceptor), requires calibration to account for polarisation cross talk and is less able to quantitate changes in FRET efficiency [15, 16].

Fluorescence lifetime measurements are independent of fluorophore concentration, excitation and detection efficiencies and the impact of scattering and sample absorption [17]. Fitting fluorescence decay profiles to an appropriate multi-exponential decay model can directly yield the FRETing fraction of the donor population and fluorescence lifetime-based FRET measurements can be readily compared across different instruments. FLIM can also be used to map other variations in local fluorophore environment, e.g. reporting on the concentration of analytes such as calcium using fluorescent dyes [18] or on physical changes such as temperature [19] or membrane lipid order [20]. Furthermore, FLIM readouts can be compared between cell-based assays and *in vivo* FRET measurements and thus potentially be translated along the drug discovery pipeline to animal models [21] since they do not require calibration. In spite of these advantages, FLIM has not yet made a significant impact on drug discovery, partly due to a lack of available FLIM instrumentation for automated multiwell plate readouts. FLIM is often implemented using laser scanning microscopes with time correlated single photon counting (TCSPC) [22–24], for which the sequential pixel acquisition of this approach typically results in data acquisition times of 10's–100's of seconds per cell to acquire sufficient detected photons, depending on the sample brightness, with the accuracy of fluorescence lifetime determination being proportional to the square root of the number of photons detected [25]. Such acquisition times are impractical for HCA and, if the excitation intensity is increased to permit much faster imaging, there are significant issues with photobleaching and phototoxicity. Recently an imaging multiwell plate reader utilising multiphoton TCSPC FLIM was reported [15] but this was for secondary measurements following a first pass by steady-state polarisation-resolved anisotropy imaging as TCSPC FLIM required 12.5 hours to image 50 wells at 5 fields of view (FOV) per well – compared to 43 minutes to image the same FOVs with polarisation.

Although TCSPC FLIM may ultimately become suitable for HCA with the development of more sensitive detectors and multichannel excitation/detection schemes, its implementations to date have encouraged a perception that FLIM HCA is not practical. We therefore believe that it is important to demonstrate the advantages and practicality of FLIM for FRET assays of PPIs and here present a quantitative evaluation of FRET assays undertaken on an optically sectioning FLIM multiwell plate reader utilising wide-field time-gated imaging that is able to automatically read a 96 well plate in 10's minutes [21] (including sample movement and automatic focusing).

Wide-field detection permits rapid imaging with significantly reduced photobleaching compared to laser scanning microscopy and can be implemented in the time domain using a gated optical image intensifier or in the frequency domain using a sinusoidally modulated optical image intensifier [26] or a CMOS camera with modulated gain [27]. Frequency domain and time domain FLIM can provide equivalent data, but there are performance trade-offs for specific implementations of both approaches. We note that the first HCA instrument for unsupervised FLIM of multiwell plate sample arrays [28] utilised wide-field frequency domain FLIM in a non-sectioning microscope, elegantly demonstrating the potential of automated FLIM-FRET and the statistical analysis of FLIM array data. Subsequently a wide-field frequency domain FLIM plate reader has been applied to image post translational modifications (tyrosine phosphorylation) *in situ* – specifically uncovering components that transduce signals from epidermal growth factor receptors [29] and impressively illustrating the potential impact of FLIM HCA when combined with automated sample preparation. These wide-field FLIM plate readers, however, did not benefit from optical sectioning, which can improve quantitative readouts by rejecting contributions from out-of-focus fluorescence.

Our realization of rapid FLIM and FRET for HCA has been implemented in the time domain using a gated optical image intensifier to provide wide-field time-gated imaging and incorporated a quasi-wide-field Nipkow (“spinning disc”) microscope unit to provide optical sectioning [30]. This approach has realised optically sectioned FLIM-FRET of live cells at up to 10 frames/second [31] and in a previous study we illustrated its effectiveness in a modified commercially available plate reader (GE Healthcare IN Cell 1000), demonstrating an assay of HIV-1 Gag aggregation in live HEK293T cells [21].

HIV-1 Gag proteins are the molecular machine responsible for orchestrating the assembly of nascent HIV-1 virions at the cell membrane [32]. Even in the absence of other viral proteins and enzymes, expression of HIV-1 Gag alone in cells results in the production of viral like particles (VLPs) that are similar to immature HIV-1 virions and are often used as a model system for the late stages in the HIV-1 life-cycle. Labeling the C-terminal of HIV-1 Gag proteins with fluorescent protein tags enables their distribution to be visualized and VLP formation can be assayed using FLIM-FRET to report on the aggregation of appropriately labelled Gag proteins (e.g. stochastically labelled with CFP and YFP). Membrane binding of HIV-1 Gag is a prerequisite for virion assembly and is driven by a ‘myristic switch’ mechanism whereby a sequestered myristic acid moiety (a co-translational modification to the Gag protein) present in Gag monomers is exposed to the solvent upon oligomerisation of two or more Gag proteins, resulting in an increase in hydrophobicity that drives membrane binding [33]. This ‘myristic switch’ mechanism provides an opportunity to interfere with virion formation and hence is a potential target for inhibitors of HIV-1 assembly.

Here we present an improved FLIM FRET assay of Gag protein aggregation, this time in HeLa cells that provide a superior biological host, and show how our automated FLIM plate reader can discriminate different biomolecular processes as we manipulate the HIV-1 Gag protein interactions through the use of myristic acid negative mutants that are unable to bind the plasma membrane [33]. Furthermore we apply an inhibitor of myristoylation (NMT inhibitor DDD85646 [34]) to permit, for the first time, a quantitative evaluation of FLIM FRET assay performance using dose response curves and calculation of *Z*′ factors. We use this metric to compare the assay performance of hetero-FRET and homo-FRET readouts of the Gag protein interactions and present the first FLIM assay of protein aggregation based on homo-FRET.

## 2. Materials and methods

### 2.1 Automated FLIM plate reader

**Figure 1:**
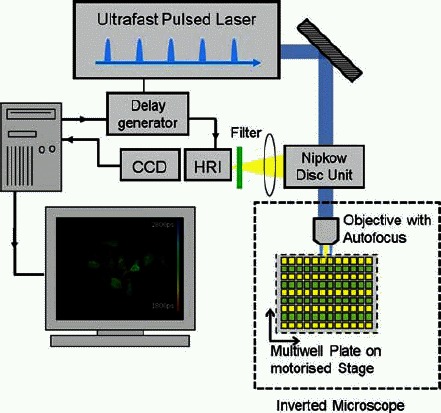
Schematic of FLIM multiwell plate reader. Tunable pulsed (∼10 ps) excitation is provided by a supercontinuum laser source coupled into the Nipkow disc unit that provides rapid scanning multipoint excitation. The resulting fluorescence passes through a spectral filter to the gated optical intensifier (HRI), which is gated at user-defined delays after the arrival of the excitation pulses and is read out using a cooled CCD camera. The motorised stage and autofocus allows fully automated location and subsequent imaging of samples.

Figure 1 presents a schematic of the automated FLIM plate reader, which is built around a wide-field microscope (Olympus IX81-ZDC) that incorporates an optical autofocus system as well as automated stage movement and changing of excitation filters. Prior to FLIM acquisition, the plate reader can perform an automatic cell finding (“pre-find”) scan to rapidly identifiy and map FOV containing suitable cells for the FLIM assay. This typically takes ∼30 minutes to find 4 FOV per well. A number of pre-find routines are available including using a lower magnification objective for increased speed and performing a pre-find in two spectral channels to ensure that only cells expressing both donor and acceptor plasmids are imaged. High speed, optically sectioned FLIM was implemented as outlined previously [30] via a Nipkow disc unit (Yokogawa Electric Corporation; CSU-X ML1) with excitation being provided by a fibre-laser pumped supercontinuum source (Fianium UK Ltd, SC 400-6) with the resultant fluorescence being detected using a gated optical intensifier (Kentech Instruments; model HRI) read out by a cooled CCD (Hamamatsu Photonics; model Orca ER II). All images were acquired using a 40× long working distance air objective (Olympus, LUCPLFLN 40×) with an NA of 0.6.

### 2.2 Cell preparation

All work was carried out using HeLa cells maintained in DMEM (Gibco) supplemented with 10% fetal calf serum (Gibco), 2.5% antibiotic and 2.5% L-glutamine. Cells were maintained at 37°C, 5% CO_2_ and grown until 80–90% confluent in T75 flasks (Corning) before passaging using trypsin to detach the cells.

Plasmids were provided by Pfizer Limited. The Gag fusion proteins were ligated into pcDNA3.1(+) expression vector (Invitrogen). Generation of the ^Myr(−)^Gag fusion proteins was achieved through the point mutation of the first *N*-terminal glycine residue within the Gag protein.

Cells were seeded in 96 well plates (Greiner, μclear) 24 hours before the start of transfections. Prior to transfection, cells were transferred from growth media, washed in phosphate buffered saline (PBS) and placed in Optimem 1 reduced serum media (Gibco). Transfections were performed using Lipofectamine 2000 (Invitrogen). Transfection mixes were prepared following manufacturer's instructions using a total of 150 ng of plasmid DNA per well with a 2:1 ratio of lipids to DNA. The transfection mixes were left on the cells for 6 hours before removal. Cells were then washed in PBS and the media replaced with growth media. The addition of NMT inhibitor doses was performed at the same time as the lipofection, with the NMT inhibitor being diluted into the Optimem 1 used to seed the cells prior to transfection. Transfections were then carried out as described above. After 24 hours, cells were washed in PBS and fixed in 4% paraformaldehyde for 15 minutes. Cells were then washed twice in PBS to remove the paraformaldehyde and imaged in PBS at room temperature.

### 2.3 Data analysis

The fluorescence decay profiles of the CFP donor were analysed by fitting the data to a single exponential decay model on a pixel-wise basis or by automatically defining regions of interest (ROIs) containing the cell membranes and fitting the binned pixels from each ROI to an exponential decay model. The fitting software is written in-house and will be described in detail in a future publication. Briefly it utilises a standard Levenberg-Marquardt non-linear least squares (NLLS) fitting algorithm incorporating reference reconvolution whereby the measurement of a short lifetime dye standard (DASPI) is used to account for the instrument response function (IRF) within the fitting algorithm.

The automatic image segmentation was performed using a routine developed in-house (MATLAB) that uses a size-tuned nonlinear top-hat function [35]. This method transforms the image pixel by pixel, enhancing the brightness of a pixel if its close vicinity is also bright and its distant vicinity is dim. A user-defined threshold is then applied to the transformed image and the resultant binary image is smoothed using standard Matlab functions and spatially distinct regions are identified. These regions are then size-sieved to remove objects smaller than a user-defined threshold. The cell segmentation masks are then eroded by a user-defined number of pixels (corresponding to the width of the cell membrane) and the the difference between the two binary images provides a binary membrane mask to apply to the FLIM data prior to fitting.

## 3. Results and discussion

**Figure 2:**
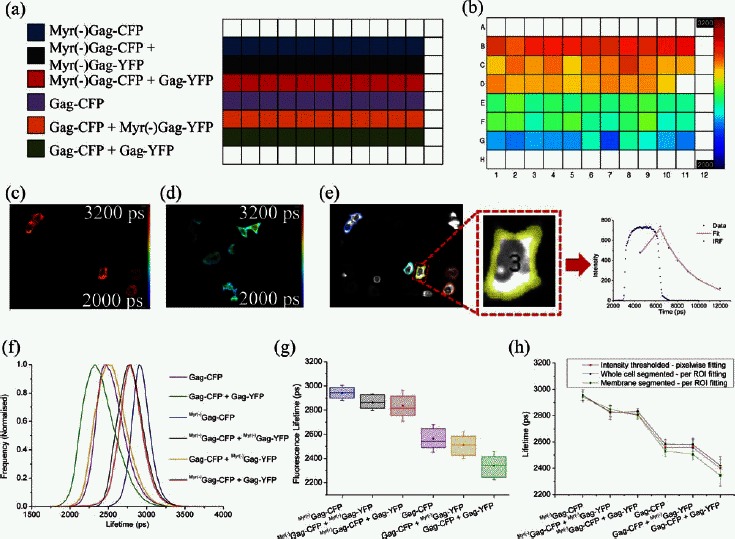
(**a**) plating scheme. (**b**) plate map of mean well lifetimes (averaged over 4 FOV per well) from pixel-wise fitting to single exponential decay model. Lifetime scale bar 3200 ps (red) to 2000 ps (blue). White squares indicate that no cells were found in that well by the automated microscope. Typical images of HeLa cells transfected with ^Myr(−)^Gag-CFP and Gag-CFP are shown in (**c**) and (**d**) respectively. (e) segmentation mask applied to FOV shown in (**c**) preferentially selecting the plasma membrane (typical ROI comprises ∼1200–1500 pixels). (**g**) box plots of lifetime ± SD for each biological construct with all ROI pixels binned and fitted to single exponential decay model on a per cell basis. (**h**) mean lifetimes per condition ± SD when data was intensity thresholded and fitted to a single exponential decay pixelwise (red). Automatic image segmentation to select cells (black) or just cell membranes (green) with the resulting data fitted using a single exponential decay model on a per ROI basis.

The automated FLIM plate reader was applied to assay HIV-1 Gag protein aggregation in HeLa cells, imaging 4 fields of view (FOV, 672 × 512 pixels corresponding to 309 × 235 mm in the image plane) of FLIM-FRET data per well, with ∼30 s/FOV average FLIM acquisition time following the pre-find scan. Figure 2 shows the results from a plate where the wells in each row were seeded with cells expressing a different preparation of Gag protein constructs, as indicated in the plate map (Figure 2a).

The lifetime data was analysed using the in-house fitting software to fit pixel-wise to a single exponential decay model, thereby providing an effective “mean lifetime” per pixel. Figure 2b is a false colour plate map of the average lifetime per well (calculated across all pixels and FOVs) and Figures 2c and d present typical optically sectioned FLIM images of HeLa cells expressing ^Myr(−)^Gag-CFP and Gag-CFP respectively (thresholded to a minimum of 200 photons acquired per pixel). Figure 2f presents the composite histograms of CFP lifetime for each of the sets of repeat wells (i.e. for each of the biological preparations). Inspection of the FLIM images indicates that higher levels of FRET (lower lifetimes) occur near the plasma membrane, as expected for VLP formation [32]. Figure 2e shows an image segmentation mask – along with an example of a single exponential decay fit of the indicated ROI – generated by applying an automatic plasma membrane segmentation algorithm to the data to select regions where the VLPs are being formed and Figure 2g presents box plots of the mean fluorescence lifetime for each biological preparation, calculated by binning the time-resolved data for each of the (near membrane) ROIs independently and fitting each to a single exponential decay model. Figure 2h displays the results from the two fitting methods on common axes. We also present results obtained when the cell segmentation algorithm was applied to segment whole cells instead of just cell plasma membranes. Differences can be seen between the application of the intensity threshold and the application of the segmentation algorithm to select entire cells. These differences arise due to inclusion of FRETing cell debris in the intensity thresholded case. This FRETing cell debris is a direct result of VLP formation and budding through the cell membrane, damaging it in the process and reducing its integrity, eventually leading to the cell breaking apart. Finally, membrane segmentation only has a noticeable effect for the constructs that are expected to aggregate, and hence FRET, at the plasma membrane of the cells, for which it increases the dynamic range of the assay.

Statistically different mean lifetimes are observed (significance level *p* < 0.05) for each of the plasmid combinations after application of an ANO-VA test with multiple mean comparisons (with the exception of ^Myr(−)^Gag-CFP + ^Myr(−)^Gag-YFP and ^Myr(−)^Gag-CFP + Gag-YFP). As expected, the mean lifetime of wild-type Gag labelled with CFP decreases (by ∼200 ps) due to FRET when co-expressed with the Gag-YFP acceptor construct compared to when it is expressed alone. The mean lifetime of the CFP attached to the Gag protein is itself lower than for the CFP attached to the mutated ^Myr(−)^Gag that does not form VLPs. This lower mean Gag-CFP lifetime can be attributed to homo-FRET between CFP fluorophores in the VLPs [36] and possibly to the different refractive index at the plasma membrane. We note that homo-FRET does not usually result in a change in fluorescence lifetime but for CFP it is thought to occur as a consequence of interactions between different states of this complex fluorophore. The prospect of a homo-FRET readout of protein aggregation is attractive since it increases the opportunities for multiplexing with other readouts as well as simplifying transfection procedures and minimising reagent use for HCA. The mean lifetime of the Gag-CFP co-expressed with mutated acceptor protein (^Myr(−)^Gag-YFP) is again slightly lower than for the Gag-CFP expressed alone. This is because, although the ^Myr(−)^Gag-YFP does not contribute to VLPs at the plasma membrane by itself, it can still dimerise with wild-type Gag-CFP protein in the cytoplasm and be ‘rescued’ into VLPs, providing acceptors for hetero-FRET. Similarly, the mutated ^Myr(−)^Gag-CFP does not form VLPs and so any FRET in the cells expressing^Myr(−)^Gag-CFP + Gag-YFP must arise from its dimerisation with Gag-YFP in the cytoplasm followed by such dimers being brought into the VLPs. Finally, the cells expressing mutated ^Myr(−)^Gag-CFP and ^Myr(−)^Gag-YFP should not form VLPs but the mean CFP lifetime is slightly lower than for the cells expressing ^Myr(−)^Gag-CFP alone, which we attribute to FRET occurring when the Gag proteins dimerise in the cytoplasm.

### 3.1 Characterisation of FLIM based HIV-1 Gag aggregation assay

This readout for Gag VLP formation can be used to assay the efficacy of compounds to inhibit HIV-1 assembly and to generate dose response curves, which themselves can be used to quantify the relative performance of assays. The enzymes N-myristoyltransferase 1 and 2 (HsNMT 1 and 2) are responsible for the co-translational addition of a myristic acid moiety to proteins within cells and compounds that block the action of these enzymes should inhibit VLP formation. We applied the NMT inhibitor DDD85646 [34] to evaluate FLIM FRET Gag VLP formation assays using hetero-FRET in cells co-transfected with Gag-CFP and Gag-YFP. We followed standardized pharmaceutical industry guidelines [37] to ensure the assay is robust and to account for experimental factors such as pipetting errors, plate edge effects, spatial uniformity and drift over time that could impact the assay quality.

The assay characterisation experiments involved the production of three 96-well plates that were prepared separately and imaged on different days to accurately replicate the situation of running a compound screen. These plates were prepared using a positive control compound (NMT inhibitor DDD85646) known to act on the target of interest, added column-wise at three different concentrations: Low (∼EC20), Medium (∼EC25-EC75) and High (∼EC80), with the order of addition changing between the plates (e.g. Plate 1 has the compound added in the repeating pattern high, medium, low and plate 2 has the compound added in the repeating pattern low, high, medium etc).

**Figure 3:**
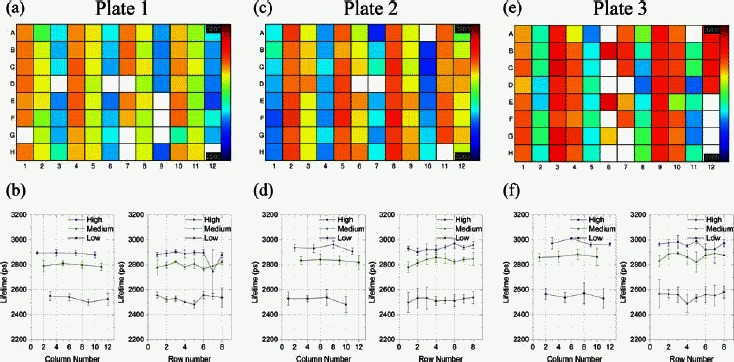
Assay characterisation plates. (**a**) & (**b**) plate map of mean well lifetimes (averaged over 4 FOV per well) from pixel-wise fitting to single exponential decay model and mean lifetimes plotted column-wise for each condition and row-wise for each condition for plate 1. (**c**) & (**d**) plate map and mean lifetime plots (column-wise and row-wise) for plate 2. (**e**) & (**f**) plate map and mean lifetime plots (column-wise and row-wise) for plate 3. White wells indicate that no cells were found by the auto find routine. Wells D6 and D7 were used for background and IRF.

Figure 3 shows the resultant lifetime plate maps from this assay characterisation study along with plots of lifetime variation for each condition either plotted column-wise (from left to right) or row-wise (from top to bottom). Data was fitted by applying an intensity threshold and fitting pixel-wise to a single exponential decay model across all acquired fields of view.

Inspection of the plate maps and plots indicates that no systematic drift or edge effects are present, showing that both the protocol used to prepare the plates and the imaging system introduce no spatial bias with regards to sample position on the plate. Slight differences in the reported lifetimes per condition are observed between the three plates for the high and medium doses of compound, but a consistent lifetime of around 2530 ps is seen for the low dose of the compound across all three plates. This indicates that the small differences between the plates are most likely to be caused by pipetting errors made during the manual preparation of the plates. This reproducibility is highlighted further when the percentage inhibition is calculated for the medium dose relative to the high and low dose for each plate, which results in inhibition percentages of 73%, 74% and 75% for plates 1, 2 and 3 respectively.

### 3.2 FLIM based dose response curves

**Figure 4:**
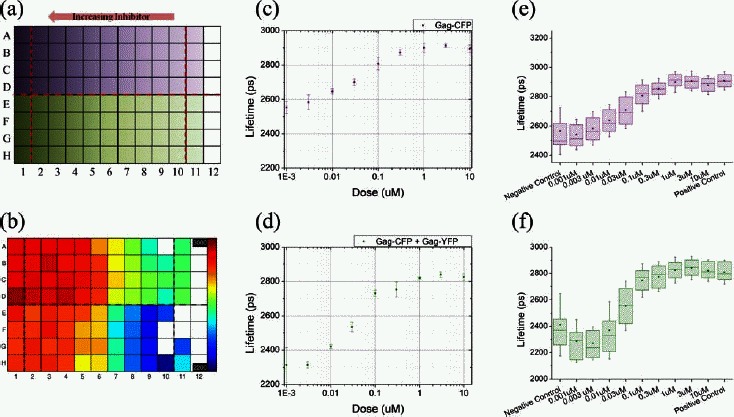
(**a**) plating scheme. Region A2-D10 contained HeLa cells transfected with WT Gag-CFP with varying amounts of NMT inhibitor (column 2: 10 μM to column 10: 0.001 μM) and region E2-H10 contained HeLa cells transfected with Gag-CFP + Gag-YFP with the same NMT inhibitor concentration range as region A2-D10. Column 1 contains HeLa cells transfected with a mutated form of the HIV Gag protein (rows A–D: ^Myr(−)^Gag-CFP, rows E–H: ^Myr(−)^Gag-CFP + ^Myr(−)^Gag-YFP) that do not form VLPs. Column 11 contains HeLa cells transfected with Gag-CFP (rows A–D) or Gag-CFP + Gag-YFP (rows E–H) that had been dosed with vehicle (DMSO) only. (**b**) mean well lifetime plate map resulting from a pixel-wise single exponential fit, lifetime scale 3000 ps (red) to 2200 ps (blue). (**c**) and (**d**) dose dependent curves (± SD) of pixel lifetimes averaged over all repeat wells for cells transfected with Gag-CFP only and Gag-CFP + Gag-YFP respectively. (**e**) and (**f**) dose dependent box plots (± SD) for pixels in segmented membrane ROIs, binned and fitted to a single exponential decay model for cells expressing Gag-CFP only and Gag-CFP + Gag-YFP respectively.

We then assayed the dose response of NMT inhibitor DDD85646 using both homo-FRET and hetero-FRET readouts by seeding a well plate with cells either expressing Gag-CFP alone or co-expressing Gag-CFP and Gag-YFP. These cells were incubated with concentrations of the NMT inhibitor varying from 10 μM to 0.001 μM (over columns 2–10), as indicated in the plate scheme in Figure 4a. Figure 4b presents the map of mean CFP lifetimes for each well (fitted to a single exponential decay model), showing the general trend of increasing lifetime (decreasing FRET) with increasing NMT inhibitor dose for both the homo-FRET (Gag-CFP only) case, and the hetero-FRET (Gag-CFP + Gag-YFP) case.

Figures 4c and d present the hetero-FRET and homo-FRET dose response curves, for which all the pixel lifetimes in the repeat wells (each well with 4 FOVs imaged, total FLIM acquisition time 3 hrs 15 mins) were averaged for each concentration of NMT inhibitor. A measure of assay performance may be obtained by calculating the *Z*′ parameter [38], which uses the differences in the mean values of the positive and negative controls and their relative spreads to generate a single value metric of assay quality. Typically a *Z*′ value of greater than 0.4 is considered desirable for a screen or assay to be useful. Here we find that for the homo-FRET assay of Gag protein aggregation with pixel-wise fitting we obtain a *Z*′ of 0.65 while for the hetero-FRET assay we find a *Z*′ of 0.35. We note, however, that for the hetero-FRET assay, our automated prefind process failed to identify any suitable cells in wells 11E and 11F and that in wells 11G and 11H it found far fewer cells than normal, which explains the lower calculated *Z*′ value for the hetero-FRET dose response. If we instead use the hetero-FRET data from colum 9 as the negative control, then we obtain a *Z*′ = 0.79, which represents a lower limit on the true value.

Figures 4e and f display box plots resulting from automatic image segmentation and binning of pixels in the membrane ROIs for all the FOV and repeat wells at each NMT inhibitor concentration – with the automatic image segmentation typically resulting in 60–100 cells per condition. At high doses of NMT inhibitor, the membrane ROI segmentation makes little difference (since there should be negligible Gag protein binding at the membrane) but at low doses this image segmentation lowers the observed mean lifetime and increases the dynamic range of the assay, resulting in *Z*′ values of 0.67 and 0.11 for the homo-FRET and hetero-FRET readouts respectively. Again the lower *Z*′ observed for the membrane segmented hetero-FRET case is due to the fact that cells were only found in two wells. As before, if we instead use column 9 as the negative control for hetero-FRET then we calculate a lower limit for *Z*′ of 0.82.

### 3.3 Estimation of dose response of aggregating Gag population fraction

We also applied global binning [39, 40], to demonstrate the potential for FLIM FRET to be used to quantify the changes in the population fraction of FRETing donors that underlies the observed changes in effective fluorescence lifetime obtained from fitting to a single exponential decay model. We applied global binning to both the Gag-CFP dose response (using ^Myr(−)^Gag-CFP as a positive control) and the co-transfected Gag-CFP & Gag-YFP dose response (using ^Myr(−)^Gag-CFP and ^Myr(−)^Gag-YFP as a positive control). In each case, τ_1_ was fixed to the value obtained from a single exponential fit of the positive control wells (column 1) to provide the non-FRETing donor lifetime. The corresponding values for τ_2_ were then determined using a double exponential fit to the globally binned decay from each dose response (transfected with Gag-CFP and co-transfected with Gag-CFP and Gag-YFP respectively), given by:


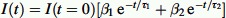
(1)

**Figure 5:**
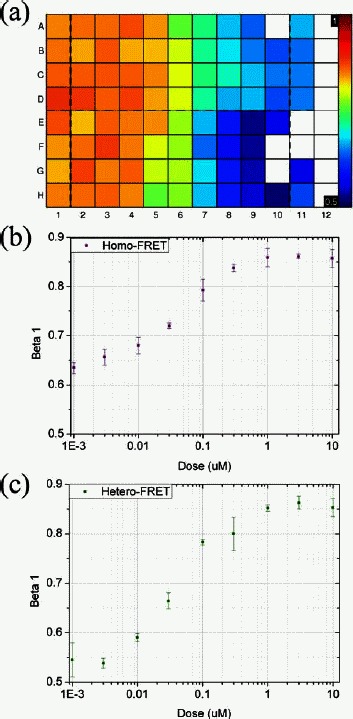
(**a**) Plate map showing the mean fraction of long lifetime component (β_1_) fitted across all FOVs for the homo- and hetero-FRET doses responses; (**b**) and (**c**) show the average β_1_ values (± SD) from all FOVs per condition for homo- and hetero-FRET dose responses. See Figure 4a for plating scheme.

CFP itself is known to have a multi-exponential decay profile but here we approximated it to a mono-exponential decay to simplify the fitting. This assumption could be avoided if a donor with a mono-exponential decay such as mTFP or mTurquoise was to be used. We also approximated the complex decay of the FRETing CFP donor observed when Gag-CFP and Gag-YFP aggregate to form VLPs to a mo-noexponential decay. The returned τ_2_ values for the homo- and hetero-FRET dose response were 766 ± 9 ps and 713 ± 25 ps respectively, where the errors are the standard deviation of values obtained by globally binning across each of the replicated rows of the plate. Figure 5a shows the *β_1_* (fraction of τ_1_) plate map for the homo- and hetero-FRET dose responses, with Figures 5b and c showing the corresponding b1 values (± SD) when averaged over repeat wells. The fraction of the long (β_1_) lifetime component (i.e. non-FRETing donor) can be seen to increase with increasing concentration of NMT inhibitor for both the homo- and hetero-FRET dose responses, as expected. This analysis illustrates the potential to use a global binning analysis of FLIM FRET data to estimate the ratio of monomer/oligomerised Gag proteins. We note this analysis would be more robust when applied to FLIM FRET data from donors with mono-exponential decays labelling proteins that dimerise but do not form larger aggregates.

## 4. Conclusion

In conclusion, we have demonstrated the application of an automated optically sectioning FLIM multiwell plate reader to assay the aggregation of HIV Gag protein during VLP formation. We have presented the first application of dose response curves and the *Z*′ parameter to evaluate the quantitative performance of FLIM assays. We have also reported the first application of FLIM-based homo-FRET readouts of protein aggregation, noting that this may be limited to complex fluorophores like CFP. The CFP homo-FRET readout has several advantages over CFP/YFP hetero-FRET including simplifying sample preparation as well as increasing spectral efficiency, which could be important for multiplexed readouts. The changes in donor (CFP) fluorescence lifetime observed in this study are relatively modest but provide robust readouts when averaged over the 96 well plate data. We note that we were not able to obtain such data on a standard TCSPC FLIM laser scanning confocal microscope (LSCM) due to the problems with photobleaching and the much smaller sample numbers – noting that it is only practical to image ∼10 FOVs per hour with our manual FLIM enabled LCSM. In contrast, our automated FLIM plate reader can image a 96 well plate in ∼10's minutes total FLIM acquisition time (at 1 FOV per well), depending on the brightness of the fluorophores expressed and the extent to which the fluorescence decays are sampled. We believe that this demonstration of the practicality of medium throughput optically sectioned FLIM HCA shows that it could aid in the optimization of lead compounds identified by HTS systems as well as providing new opportunities for basic research in cell biology, particularly in the area of PPIs. The ability to translate FLIM readouts from automated plate readers to live disease models may also have a positive impact on the drug discovery pipeline. In the immediate future we will utilise this new capability to screen for other novel inhibitors of HIV formation with a view to developing therapies for HIV and other viral infections.

## Supplementary material

As a service to our authors and readers, this journal provides supporting information supplied by the authors. Such materials are peer reviewed and may be re-organized for online delivery, but are not copy-edited or typeset. Technical support issues arising from supporting information (other than missing files) should be addressed to the authors.
