# Supplementary material for: Automated fluorescence lifetime imaging plate reader and its application to Förster resonant energy transfer readout of Gag protein aggregation
Source: J Biophotonics. 2012 Nov 27;6(5):398–408. doi: 10.1002/jbio.201200185 (PMC3660788; doi:10.1002/jbio.201200185)
Supplement: Supplementary file 1 [file jbio0006-0398-SD1.pdf]

Dominic Alibhai received a M.Sci. degree in Physics from Kings College London in 2008 and an MRes in Chemical Biology of Health and Disease from Imperial College London in 2009. He is currently pursuing a Ph.D. in the Photonics group of Imperial College London and is a member of the Institute of Chemical Biology at Imperial. His research includes the application of fluorescence lifetime imaging microscopy to the study of HIV-1 Gag protein aggregation and the development of high content based FLIM assays.

Douglas Kelly received an MPhys degree in Physics from the University of Oxford in 2009 and an MRes in Chemical biology of health and disease from Imperial College London in 2010. He is currently pursuing a Ph.D. in the Photonics group of Imperial College London and is a member of the Institute of chemical biology at Imperial. His research includes the development of instrumentation for high-throughput FLIM, and subsequent application to a range of biological systems. These encompass imaging of both cellular and tissue autofluorescence, and FRET imaging using various biosensors.

Sean Warren received an M.Sci. degree in Physics from the University of Cambridge in 2008 and an MRes in Chemical biology of health and disease from Imperial College London in 2010. He is currently pursuing a Ph.D. in the Photonics group of Imperial College London and is a member of the Institute of Chemical Biology at Imperial. His research includes the application of FLIM to study signalling processes in cells undergoing chemotaxis and the development of analysis algorithms for FLIM data.

Sunil Kumar completed a BSc degree in Physics in 2002, an MSc in Optics and Photonics in 2004, an MSci in Protein and Membrane Chemical Biology in 2005 and a PhD in 2009 at Imperial College London. Since then, he has worked on fluorescence lifetime imaging in a variety of contexts, primarily high-content screening, and also on Oblique Plane Microscopy for high-speed 3D imaging.

Anca Margineanu received a Medical Doctor degree from the University of Medicine and Pharmacy Iasi, Romania, and then a PhD in Chemistry from the Catholic University Leuven, Belgium in 2006. She is currently working as a postdoctoral research assistant in the Photonics group at Imperial College London. Her research topics include biophysics of lipid bilayers and plasma membranes, applications of new fluorescent dyes for biology, as well as FRET-FLIM and polarisation techniques to monitor molecular interactions within signalling pathways.

Remigiusz Serwa is currently a Marie Curie Research Fellow in Chemical Biology in the Department of Chemistry at Imperial College London. He obtained a PhD in Chemistry from Vanderbilt University (Nashville TN, USA) and worked as a postdoctoral scientist at the Free University of Berlin. For over five years the main focus of his research has been the development of chemical tools for studying post-translational modifications of proteins.

Emmanuelle Thinon received a M.Sci. degree in Organic Chemistry from Strasbourg University, France in 2009. She is currently pursuing a Ph.D. in Chemical Biology at Imperial College London. Her research focuses on the identification of novel inhibitors for N-myristoylation and includes the study of myristoylation in cancer cells.

Yuriy Alexandrov holds a Ph.D. degree in Applied Physics from Hebrew University of Jerusalem. He is currently a Research Assistant in the Photonics group at Imperial College London. Yuriy's research includes image and signal processing for FLIM applications, software design, and analysis of biomedical data.

Ted Murray received his PhD from University of London in 1985 for epigenetic studies on DNA methylation and regulation of human globin gene expression. He then used viral systems as models for mammalian gene regulation and more recently used viral replication systems to develop antiviral therapies at Roche and Pfizer research laboratories. He is now senior virologist at Retroscreen Virology Limited.

Frank Stuhmeier received a PhD degree in Physical Chemistry from the University of Göttingen (Germany) in 1998 for experimental work carried out at the Max-Planck-Institute for Biophysical Chemistry. From 1998 to 1999 he was a postdoctoral researcher at the Laboratory for Fluorescence Dynamic at the University of Illinois at Urbana-Champaign (USA). He joined Pfizer Worldwide Research in 1999 and worked as a Drug Discovery Scientist until 2011 before moving into Clinical Development.

Edward W. Tate is currently a Reader in Chemical Biology in the Department of Chemistry and Institute of Chemical Biology, Imperial College London. He received his PhD in organic chemistry in the group of Prof Steve Ley FRS at the University of Cambridge, and then undertook postdoctoral research in chemistry at Ecole Polytechnique (Paris) as an 1851 Research Fellow, and in molecular microbiology at the Pasteur Institute in Paris. He then moved to Imperial College London, where he was awarded a BBSRC David Phillips Research

Fellowship in 2006 to start an independent research group focussing on the design and application of chemical approaches to understanding living systems, with an emphasis on the roles of protein modification in disease.

Chris Dunsby received an MSci. degree from Bristol University in 2000. In 2003 he received a Ph.D. from Imperial College, UK, in “Wide-field Coherence-gated Imaging Techniques Including Photorefractive Holography”. He is now a joint lecturer between Photonics, Department of Physics and the Department of Histopathology, Division of Investigative Science at Imperial. His research interests are centred on the application of photonics and ultrafast laser technology to biomedical imaging and include multiphoton microscopy, multi-parameter fluorescence imaging and fluorescence lifetime imaging.

Mark Neil received a Ph.D. in Optical Information Processing at the University of Cambridge. In 1989 he moved to the Department of Engineering Science at Oxford University where he worked on holographic and integrated optic components in photochromic materials and ferro-electric spatial light modulators as programmable phase devices for diffractive optics applications. Since 1996 he has also been working on advanced microscopy techniques and applications and is co-inventor of several techniques for realising optical sectioning in wide field microscopes. In 2002 he joined the Photonics Group in the Department of Physics at Imperial College London where he is Professor of Photonics.

Paul French received the B.Sc. Degree in Physics in 1983 and the Ph.D. degree (for work on femtosecond dye lasers) in 1987 from Imperial College London. Much of his subsequent research career has been based at Imperial College London where he has concentrated on ultrafast laser technology and its applications, particularly in biomedical optics. In 1988 he was a visiting professor at the University of New Mexico and from 1990 to 1991 he worked on ultrafast all optical switching in optical fibres at AT&T Bell Laboratories, Holmdel, NJ. He is a Professor of Physics at Imperial College London and is Head of the Photonics Group. His current research includes a strong emphasis on fluorescence lifetime imaging for molecular biology, drug discovery and clinical applications. Prof. French is a Fellow of the Institute of Physics, the European Physical Society and the Optical Society of America and was the recipient of a Royal Society Wolfson Research Merit Award.
